# A nitroalkene derivative of salicylate alleviates diet-induced obesity by activating creatine metabolism and non-shivering thermogenesis

**DOI:** 10.21203/rs.3.rs-3101395/v1

**Published:** 2023-07-12

**Authors:** Karina Cal, Alejandro Leyva, Jorge Rodríguez-Duarte, Santiago Ruiz, Leonardo Santos, Lucía Colella, Mariana Ingold, Cecilia Vilaseca, German Galliussi, Lucía Ziegler, Thais R. Peclat, Mariana Bresque, Rachel M Handy, Rachel King, Larissa Menezes dos Reis, Camila Espasandin, Peter Breining, Rosina Dapueto, Andrés Lopez, Katie L. Thompson, Guillermo Agorrody, Evan DeVallance, Ethan Meadows, Sara E. Lewis, Gabriele Catarine Santana Barbosa, Leonardo Osbourne Lai de Souza, Marina Santos Chichierchio, Valeria Valez, Adrián Aicardo, Paola Contreras, Mikkel H. Vendelbo, Steen Jakobsen, Andrés Kamaid, Williams Porcal, Aldo Calliari, José Manuel Verdes, Jianhai Du, Yekai Wang, John M Hollander, Thomas A. White, Rafael Radi, Guillermo Moyna, Celia Quijano, Robert O’Doherty, Pedro Moraes-Vieira, Graham P Holloway, Roberta Leonardi, Marcelo A Mori, Juliana Camacho-Pereira, Eric E. Kelley, Rosario Duran, Gloria V. Lopez, Carlos Batthyány, Eduardo N. Chini, Carlos Escande

**Affiliations:** 1- Laboratory of Metabolic Diseases and Aging, Institut Pasteur Montevideo, Uruguay; 2- Laboratory of Vascular Biology and Drug Development, Institut Pasteur Montevideo, Uruguay; 3- Unidad de Bioquímica y Proteómica Analíticas, Institut Pasteur de Montevideo, IIBCE, Uruguay.; 4- Departamento de Fisiología, Facultad de Medicina, Udelar, Uruguay.; 5- Laboratory of Immunoregulation and Inflammation; Institut Pasteur Montevideo, Uruguay; 6- Departamento de Ecología y Gestión Ambiental, Centro Universitario Regional del Este, Udelar, Maldonado, Uruguay; 7- Mayo Clinic Robert and Arlene Kogod Center on Aging, Mayo Clinic, Rochester, MN, USA; 8- Department of Anesthesiology, Mayo Clinic, Rochester, MN, USA; 9- Department of Physiology and Biomedical Engineering; Mayo Clinic, Rochester, MN, USA; 10- Department of Human Health and Nutritional Sciences, University of Guelph, Guelph, Ontario, Canada; 11- Department of Biochemistry and Molecular Medicine, West Virginia University, Morgantown WV, USA.; 12- Laboratory of Immunometabolism, Department of Genetics, Evolution, Microbiology, and Immunology, Institute of Biology, University of Campinas, SP, Brazil; Department of Immunology, Institute of Biomedical Sciences, University of São Paulo, SP, Brazil; Obesity and Comorbidities Research Center (OCRC), University of Campinas, SP, Brazil; Experimental Medicine Research Cluster (EMRC), University of Campinas, SP, Brazil.; 13- Unidad Bioquìmica, Facultad de Veterinaria, Udelar, Uruguay; 14- Department of Biomedicine, Aarhus University, Denmark; 15- Área I+D Biomédico, CUDIM, Uruguay; 16- Laboratorio de Fisicoquímica Orgánica, Departamento de Química del Litoral, CENUR Litoral Norte, Udelar, Uruguay.; 17- Departamento de Fisiopatología, Hospital de Clínicas, Facultad de Medicina, Udelar, Uruguay.; 18- Department of Physiology and Pharmacology, School of Medicine, West Virginia University, Morgantown, WV, USA; 19- Mitochondria, Metabolism and Bioenergetics Working Group; School of Medicine, West Virginia University, Morgantown, WV, USA; 20- Laboratory of Bioenergetics and Mitochondrial Physiology, Institute of Medical Biochemistry Leopoldo de Meis, Federal University of Rio de Janeiro, Brazil.; 21- Cátedra de Bioquímica y Biofísica, Facultad de Odontología, Udelar, Uruguay.; 22- Centro de Investigaciones Biomédicas (CEINBIO), Udelar, Uruguay.; 23- Departamento de Bioquímica, Facultad de Medicina, Udelar, Uruguay.; 24- Departamento de Nutrición Clínica, Escuela de Nutrición, Udelar, Uruguay.; 25- Department of Nuclear Medicine and PET, Aarhus University Hospital, Denmark; 26- Unidad de Bioimagenología Avanzada. Institut Pasteur de Montevideo, Uruguay; 27- Departamento de Química Orgánica, Facultad de Química, Udelar, Uruguay.; 28- Unidad Biofísica, Departamento de Biociencias, Facultad de Veterinaria, Udelar, Uruguay; 29- Unidad Patología, Departamento de Patobiología; Facultad de Veterinaria, Udelar, Uruguay; 30- Department of Ophthalmology and Visual Sciences, Department of Biochemistry, West Virginia University, Morgantown, USA; 31- Division of Exercise Physiology, West Virginia University, Morgantown, USA; 32- Department of Medicine, Division of Endocrinology and Metabolism, University of Pittsburgh, Pennsylvania.; 33- Department of Microbiology and Molecular Genetics; University of Pittsburgh, Pennsylvania.; 34- Department of Biochemistry and Tissue Biology, Institute of Biology, University of Campinas, SP, Brazil; Obesity and Comorbidities Research Center (OCRC), Campinas, SP, Brazil; Experimental Medicine Research Cluster (EMRC), Campinas, SP, Brazil; Instituto Nacional de Obesidade e Diabetes, Campinas, SP, Brazil.; 35- Center for Inhalation Toxicology (iTOX), School of Medicine, West Virginia University, Morgantown, USA.; 36- Department of Anesthesiology and Perioperative Medicine, Mayo Clinic, Jacksonville, Florida, USA.

**Keywords:** Adipose tissue, obesity, creatine, thermogenesis, energy expenditure, salicylate derivative

## Abstract

Obesity-related type II diabetes (diabesity) has increased global morbidity and mortality dramatically. Previously, the ancient drug salicylate demonstrated promise for the treatment of type II diabetes, but its clinical use was precluded due to high dose requirements. In this study, we present a nitroalkene derivative of salicylate, 5-(2-nitroethenyl)salicylic acid (SANA), a molecule with unprecedented beneficial effects in diet-induced obesity (DIO). SANA reduces DIO, liver steatosis and insulin resistance at doses up to 40 times lower than salicylate. Mechanistically, SANA stimulated mitochondrial respiration and increased creatine-dependent energy expenditure in adipose tissue. Indeed, depletion of creatine resulted in the loss of SANA action. Moreover, we found that SANA binds to creatine kinases CKMT1/2, and downregulation CKMT1 interferes with the effect of SANA *in vivo*. Together, these data demonstrate that SANA is a first-in-class activator of creatine-dependent energy expenditure and thermogenesis in adipose tissue and emerges as a candidate for the treatment of diabesity.

## Graphical Abstract

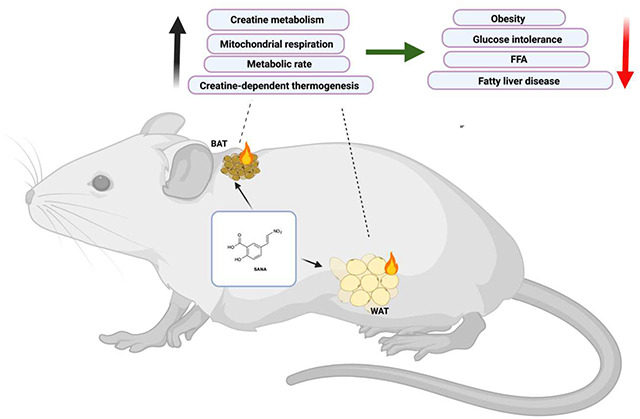

## Introduction

Obesity and its comorbidities, including type II diabetes and cardiovascular disease^1^ are pandemic and thus a prime driver of healthcare burden worldwide. Whereas obesity is preventable via adoption of healthy nutrition and lifestyle, once the obese phenotype has been established it is difficult to reverse by behavioral changes. Therefore, there is a pressing need for developing novel pharmacologic strategies for prevention and treatment of obesity.

Salicylate, an ancient drug, and precursor of aspirin was used for more than a century to diminish inflammation and relieve pain. Salicylate (and aspirin) inhibits NFkB signaling and inflammation^2^. When administered at elevated doses (2–5 g/kg/day), it activates AMPK and improves metabolic dysfunction in obese mice, including modest effects in weight gain^3–6^. This salutary action combined with its safety profile, incentivized clinical trials to treat type II diabetes and cardiovascular disease. While these trials revealed improvement in basal glycemia, glycosylated hemoglobin and CRP levels, they were discontinued due to the excessive dose required (up to 4 g/day) and the modest beneficial outcomes^7–9^. Nevertheless, the widespread use of salicylate, especially in the last century in the form of aspirin which is consumed daily by half of the adult population in the USA^10^, provides a safety underpinning for the development of novel, affordable, safe, and more effective salicylate-derived drugs.

Like salicylate, nitro fatty-acids have demonstrated pleiotropic salutary beneficial properties^11–14^, including beneficial metabolic effects during obesity^13–15^. Recently, we showed that the nitroalkene group can be moved to molecular scaffolds with known pharmacological effects that are structurally different from fatty acids. Re-localization of the nitroalkene group to these molecules allowed us to establish three concepts. First, the beneficial signaling properties of the nitroalkene group were preserved in the new molecules. Second, the accepting scaffold could play a role in improving the pharmacological properties of the nitroalkene group; and third, in some cases there are emergent properties in the new molecules that could not be predicted before, probably due to the combined pharmacological effect of the nitroalkene group and the scaffold. These findings led us to show that novel nitroalkene-containing molecules were efficient in pre-clinical models of different diseases, such as ALS^16^, atherosclerosis^17^, and glucose intolerance during obesity^18^. Based on these concepts and taking into consideration the well-known actions of salicylate on inflammation and metabolism regulation^6–9,19^ we designed a nitroalkene derivative of salicylate, 5-(2-nitroethenyl)salicylic acid (SANA) and assessed its metabolic effects in murine DIO. Our data demonstrate that SANA promotes weight loss and protects against DIO, insulin resistance, hypertriglyceridemia, and liver steatosis. Notably, these effects are markedly greater than those obtained with salicylate, using effective doses up to forty times lower. Mechanistically, we found that SANA stimulates creatine metabolism and non-shivering thermogenesis in adipose tissue, increasing energy expenditure. Untargeted proteomics identified CKMT1/2 as molecular targets of SANA. *Ckmt1* KO mice demonstrated impaired thermoregulation after SANA treatment, that was rescued by thermoneutral housing. SANA is a first-in-class activator of creatine-dependent energy expenditure and non-shivering thermogenesis in adipose tissue, providing evidence that stimulation of this pathway may be suitable for the treatment of obesity and its metabolic complications.

## Results

### The synthetic salicylate derivative SANA (5-(2-nitroethenyl)salicylic acid) prevents diet-induced obesity.

**Figure 1.**
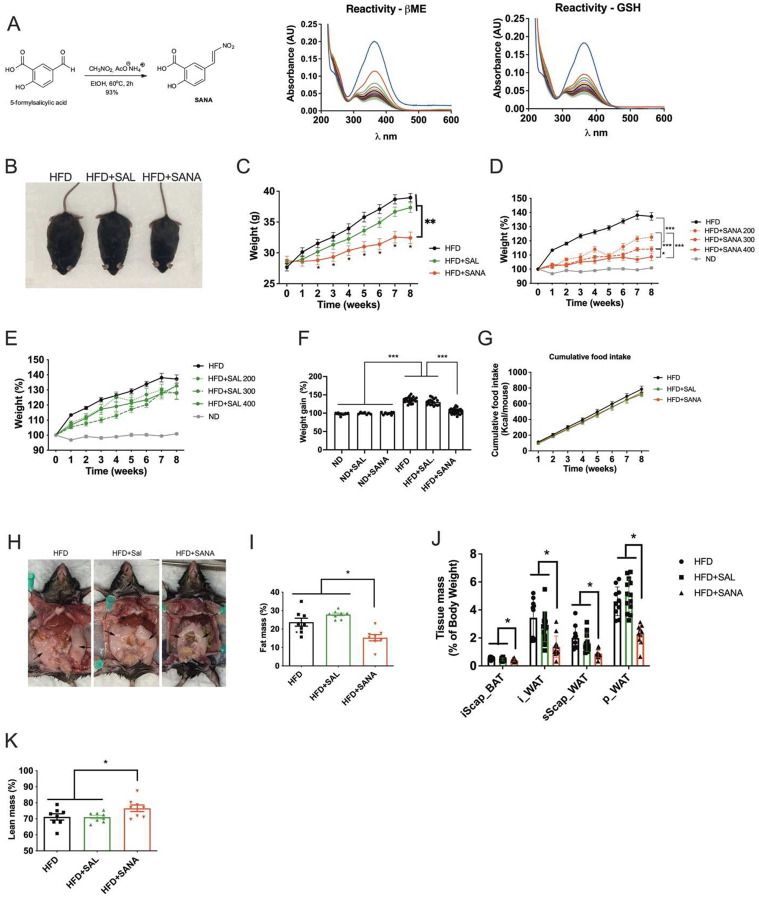
SANA protects against diet-induced obesity. **A)** SANA synthesis route (left) and characterization of its electrophilic properties (right). SANA (10 μM) was incubated with β-Mercaptoethanol (BME, 100 μM) or reduced glutathione (GSH, 100 μM). Spectra of the reaction were obtained in the 200–600 nm range every 60 s. **B)** Representative picture of mice fed with high-fat diet (HFD) or HFD+SANA (or salicylate, SAL) at 400 mg/kg/day (PO) **C)** Weight gain of the mice shown in B). **D-E)** Weight gain expressed as percent of initial weight in mice in normal diet (ND) or fed with HFD alone or supplemented with different doses of SANA (in **D**) or SAL (in **E**). Compounds were administered PO at 200, 300 and 400 mg/kg/day. **F)** Percent of weight gain in mice fed with ND alone and ND+SANA (or SAL) at 400 mg/kg/day after 4 weeks of treatment or HFD+SANA (or SAL) at 400 mg/kg/day after 8 weeks. **G)** Cumulative food intake in mice treated as described in B.**H)** Representative picture of mice treated as described in B. Arrows point to perigonadal fat depots. **I)** Total fat mass was measured by EchoMRI and **J)** Quantitation of different fat depots of mice treated as described in B). Brown adipose tissue (iscap_BAT), inguinal subcutaneous (i_WAT), subscapular subcutaneous (sScap_WAT) and perigonadal (p_WAT) white adipose. **K)** Total lean mass was measured by EchoMRI in the same conditions described in B).

The salicylate-based nitroalkene, 5-(2-nitroethenyl)salicylic acid (SANA), was obtained by a one-step synthetic route with ~93% yield (Fig. 1A). SANA showed similar biochemical (i.e., Michael addition reactions with low molecular weight thiol-containing compounds, Fig. 1A) and signaling properties to other nitroalkene compounds (**Supplementary Fig. 1**). Interestingly, SANA demonstrated protection from diet-induced obesity (DIO) (Fig. 1B–F), despite not affecting total food consumption (Fig. 1G). Importantly, SANA showed a maximum effect on obesity at doses where salicylate was ineffective (Fig. 1C–F). HPLC and MS analysis from plasma showed that the main metabolite was the saturated form of the molecule 5-(2-nitroethil)salicylic acid (M1) (**Supplementary Fig. 1**). Treatment of mice with 5-(2-nitroethil)salicylic acid had no effect on DIO and glucose management, confirming the need of the reactive nitroalkene group for the metabolic effect of SANA **(Supplementary Fig. 1).** Interestingly, treatment of mice with (E)-4-(2-nitrovinyl) benzoic acid (BANA), a nitroalkene benzoic acid derivative (that only lacks the hydroxyl group of the benzoic ring) and has well-documented protective actions in ALS disease models^16^ had no effect on DIO and glucose homeostasis **(Supplementary Figure 1)**, suggesting that that the nitroalkene group is necessary but not sufficient, and highlighting the role of the combination of the scaffold plus the nitroalkene group in the pharmacological effect of SANA. Protection against DIO by SANA was due to decreased fat accumulation in all adipose depots analyzed (Fig. 1H–J), paralleled by an increase in the percent of lean mass in the SANA-treated mice (Fig. 1K).

### SANA protects mice from DIO-associated glucose intolerance and fatty liver disease.

**Figure 2.**
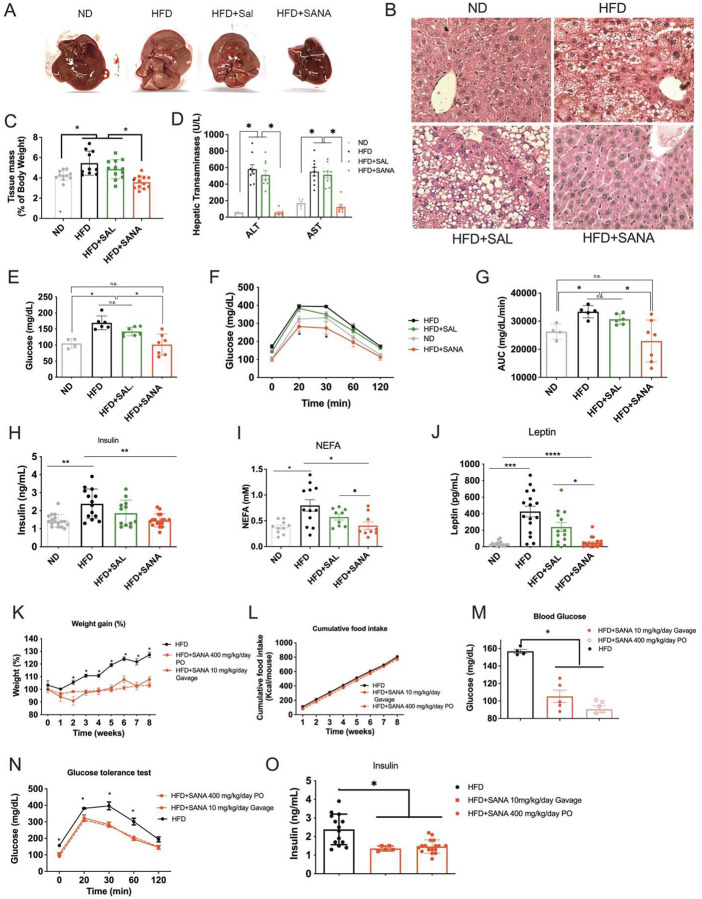
SANA protects against glucose intolerance and liver steatosis in response to DIO. **A-C)** Liver macroscopic appearance (A), H&E liver staining (B) and liver weight (C) in mice fed with ND, HFD, HFD+SANA or SAL at 400 mg/kg/day (PO). **D)** Liver transaminases in plasma/serum and **E-G)** Fasting glucose, glucose tolerance test (GTT) and GTT area under the curve (AUC) in mice treated as described in C). **H-J)** Quantitation of insulin (H), NEFA (nonesterified fatty acids) (I) and Leptin (J), in plasma/serum from mice treated as described in C). **KO)** SANA was delivered orally in solution (by gavage) at 10 mg/kg/day and compared with the dose of 400 mg/kg/day PO. **K)** Weight gain. **L)** Cumulative food intake, **M)** Fasting glucose levels at week 8. **N)** GTT at week 8. **O)** Insulin plasma levels at week 8.

While assessing the metabolic consequences of DIO, we found that livers from HFD mice treated with SANA had normal macroscopic appearance, indistinguishable from lean, age-matched control mice (Fig. 2A). Histologic analysis of livers confirmed these findings, revealing that mice treated with SANA showed no signs of liver steatosis (Fig. 2B). Examination of livers revealed SANA treatment abrogated DIO-mediated elevation in total liver weight induced by HFD (Fig. 2C). Analysis of liver transaminases in plasma showed complete protection from obesity-related liver damage (Fig. 2D). Assessment of glucose management after DIO revealed that SANA protected mice from elevated blood glucose levels (Fig. 2E). Furthermore, SANA improved impaired glucose tolerance (GTT) and made it indistinguishable from lean age-matched controls and significantly better than obese mice treated with salicylate (HFD+SAL) (Fig. 2F–G). Obese mice treated with SANA showed improved insulin, free fatty acid (FFA) and leptin levels (Fig. 2H–J). AMPK activation by salicylate and SANA occurred at similar doses both *in vitro* and *in vivo* **(Supplementary Fig. 2)**, indicating that enhanced AMPK activation by SANA is unlikely to account for most of its effects, thus suggesting other mechanisms might be operative. Biodistribution analysis performed by ^11^C-SANA followed by PET-MRI scan revealed detectable SANA in inguinal white adipose tissue (**Supplementary Fig. 2**). Importantly, the optimized formulation of SANA via complete solubilization and pH-dependent stabilization afforded capacity to achieve tissue concentrations like oral powder administration. Doses as low as 10–20 mg/kg/day provided concomitant protective effects on weight gain, fasting blood glucose levels and glucose tolerance (Fig. 2K–O, 10 mg/kg/day, gavage, **and Supplementary Fig. 2,** 20 mg/kg/day, SC).

### SANA is effective in treating obesity, glucose intolerance and liver steatosis.

**Figure 3.**
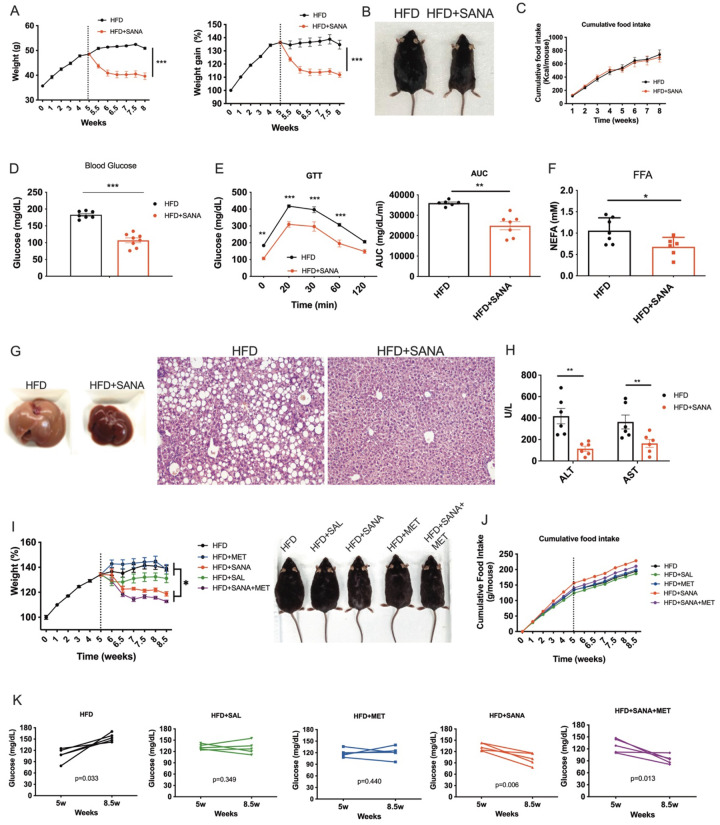
Treatment of obese mice with SANA promotes weight loss and amelioration of glucose intolerance and liver damage. Obese mice were treated with SANA at 200 mg/kg/day **PO. A)** Weight gain and percent of initial weight. **B)** Representative picture and **C)** Cumulative food intake. **D)** Fasting glucose levels measured at week 8. **E)** GTT at week 8. **F)** Free-fatty acids levels in plasma at week 8. **G)** Liver macroscopic appearance and H&E liver staining and **H)** Liver transaminases in plasma/serum from mice at week 8. **I)** Percent of initial weight and representative picture of obese mice treated with SANA or SAL (200 mg/kg/day PO), in combination or not with metformin (MET 300 mg/kg/day, gavage). **J)** Cumulative food intake and **K)** evolution of fasting glucose.

Addressing DIO and metabolic complications once they are established is more representative of the current clinical need in humans. As such, SANA was administered after obesity was established (5 weeks of HFD). SANA-treated mice lost weight (Fig. 3A–C), demonstrated diminished basal glycemia (Fig. 3D), improved glucose tolerance (Fig. 3E), and showed no signs of hepatic steatosis (Fig. 3F–H). Importantly, SANA (200 mg/kg/day) not only promoted weight loss, but also showed increased effectiveness compared to salicylate (200 mg/kg/day) and metformin (300 mg/kg/day) in glycemic control during the 3-week period studied (Fig. 3I–K). No signs of toxicity were found during the treatments. Water consumption, stools and urine production were not affected by SANA **(Supplementary Fig.3).** Analysis of renal histology and function (creatinine clearance) showed no signs of kidney damage **(Supplementary Fig. 3)**. Indeed, continuous administration of SANA for up to twenty-five weeks resulted in not a single mortality case in mice (n=15 per experimental group), highlighting not only the efficacy but also the safety of the compound at the tested doses.

### Proteomic analysis from inguinal white adipose tissue (iWAT) and mitochondria shows a signature of creatine metabolism and non-shivering thermogenesis in response to SANA.

**Figure 4.**
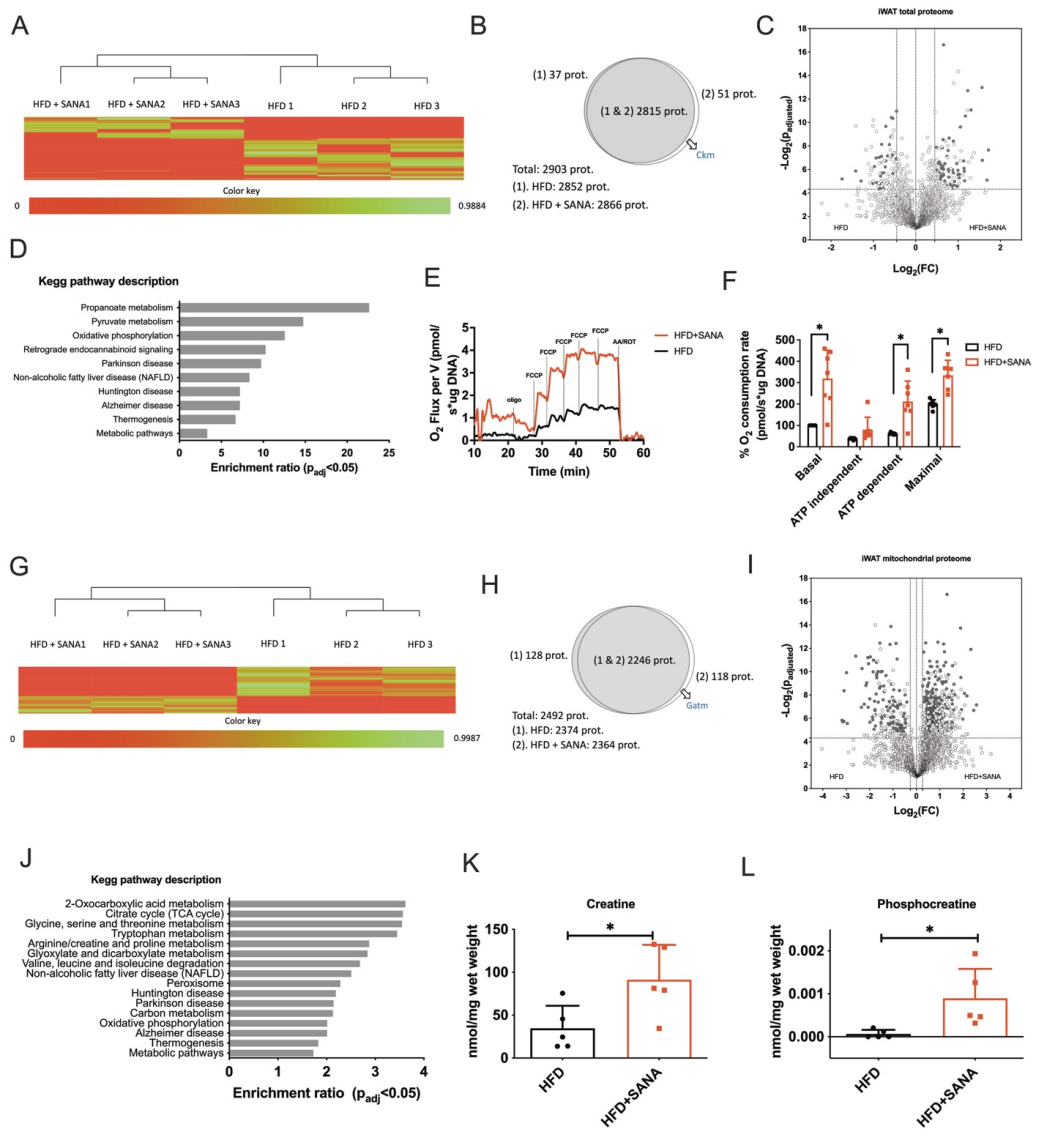
Proteomic analysis of whole tissue and isolated mitochondria from iWAT to SANA. **A-D)** Whole iWAT proteomic analysis from obese mice fed with HFD or HFD+SANA (400 mg/kg/day, PO). **A)** Heatmap generated showing an individual protein per row and biological replicates of each condition in columns. **B)** Venn diagram indicating the proteins exclusively detected in each condition (p-value < 0.05). Ckm: Creatine kinase M-type. **C)** Volcano plot showing proteins found in both conditions with statistically differential abundance (BH q-value < 0.05). Each dot represents a protein detected in at least 4 biological replicates from the total of 6 in both conditions. The darkest dots correspond to statistically differential proteins. **D)** WebGestalt’s pathway over-representation analysis of proteins overexpressed in HFD+SANA vs HFD. **E)** Cellular respiration and **F)** oxygen consumption in white adipocytes isolated from mice treated as described in A-D). **G-J)** Proteomic analysis of isolated mitochondria from iWAT of mice treated as described in A-D). Gatm: Glycine amidinotransferase, mitochondrial. **K)** Total creatine and **L)** phosphocreatine levels in iWAT from mice treated as described in A-D), measured by MS.

The phenotype triggered by SANA pointed to increased energy expenditure in adipose tissue as a putative mechanism of action. Based on the recent emergence of novel thermogenic pathways, we used unbiased proteomic analysis in iWAT as a discovery approach. Label-free quantitative proteomic analysis in iWAT of mice revealed a consistent shift in the pattern of protein expression between obese mice and obese mice treated with SANA (Fig. 4A). Proteins uniquely detected in HFD or HFD+SANA, as well as those differentially abundant between conditions were pinpointed (Fig. 4B and C; **Supplementary Tables I and II**). Combined, 109 proteins were increased in HFD+SANA iWAT tissue. KEGG pathway enrichment analysis showed that SANA stimulated catabolism, oxidative phosphorylation, and thermogenesis (Fig. 4D; **Supplementary Table III**). Isolated adipocytes from mice treated with SANA demonstrated increased mitochondrial respiration (Fig. 4E–F), consistent with the signature from tissue proteomic data. SANA had no direct effect on respiration in isolated brain or brown adipose tissue mitochondria *in vitro* **(Supplementary Fig. 4–5)** or in non-adipose cells in culture **(Supplementary Fig. 4)**. Next, we extended the proteomic analysis to isolated mitochondria from iWAT. Heatmap and differential analyses from mitochondrial proteomes showed a dramatic change in the protein expression profile among treatments (Fig. 4G–I; **Supplementary Tables IV and V**). In this case, 380 proteins were identified as statistically overrepresented in HFD+SANA (Fig. 4H–I). Pathway enrichment analysis showed that thermogenic, metabolic and TCA cycle, arginine-, glycine- and proline-related metabolic pathways were up-regulated by SANA (Fig. 4J; **Supplementary Table VI**). These results resembled those previously described^20^ after cold-induced thermogenesis in mice and pointed to creatine-dependent thermogenesis. Interestingly, among the proteins detected only after SANA treatment, GATM (Glycine amidinotransferase), the rate-limiting step in creatine synthesis (Fig. 4H), and the creatine kinase CKM were present (Fig. 4B). Consistent with the metabolic signature of creatine-dependent thermogenesis described previously^20^ and the up-regulation of GATM (Fig. 4H), we found a significant increase in creatine and P-creatine levels in iWAT from SANA-treated mice (Fig. 4K–L).

### SANA stimulates thermogenesis in the absence of UCP1 activation, an effect that is abolished when creatine metabolism is impaired *in vivo*.

**Figure 5.**
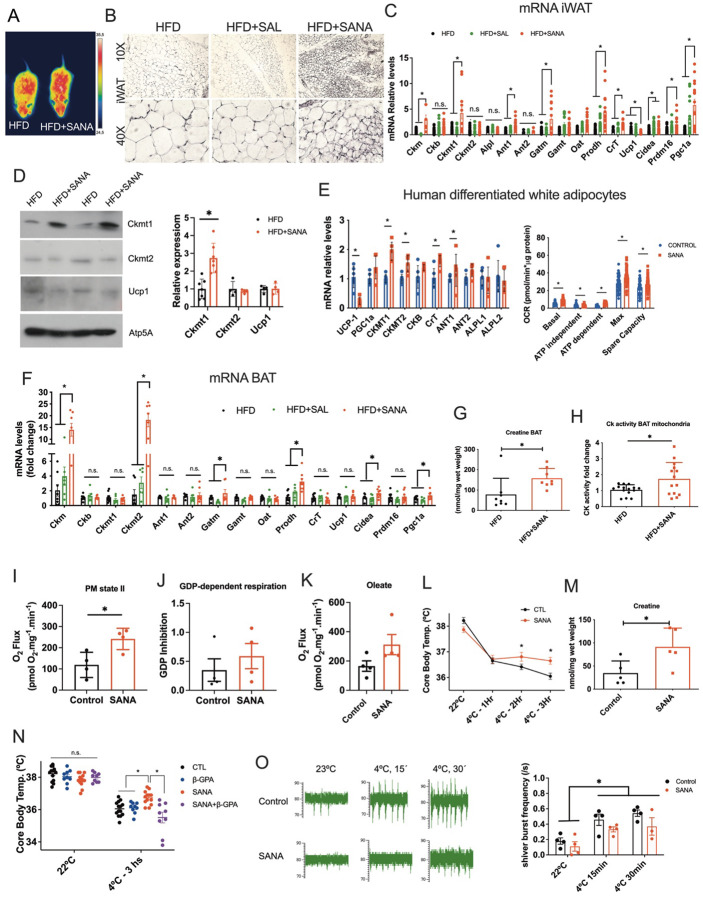
SANA stimulates thermogenesis in the absence of UCP1 activation, an effect that is abolished when creatine metabolism is impaired *in vivo*. **A)** Thermal image of mice fed with HFD or HFD+SANA (400 mg/kg/day, PO). **B-D)** Analysis of inguinal white adipose tissue **B)** Representative histological (H&E) image of iWAT. **C)** Expression of thermogenesis markers by qPCR in iWAT from mice treated as described in A. **D)** WB analysis of CKMT1, CKMT2, UCP1 in iWAT after SANA treatment as described in A. **E)** Human differentiated white adipose cells (TERT-hWA) were incubated with SANA (100 μM) for 24 hours. Left, expression of thermogenic markers measured by qPCR. Right, mitochondrial respiration. **F-K)** Analysis of brown adipose tissue (BAT). **F)** Expression of thermogenesis markers by qPCR in BAT, including scaffold as a further control; G**)** total creatine levels in BAT measured by MS and **H)** creatine kinase activity in BAT isolated mitochondria, from mice treated as described in A. **I)** State II respiration in isolated mitochondria from BAT measured with pyruvate+malate (PM). **J)** Inhibited respiration of isolated mitochondria from BAT by GDP **K)** FFA-dependent respiration of isolated mitochondria from BAT after UCP1 inhibition with GDP. **L)** Cold challenge from mice treated with SANA (20 mg/kg/day, SC) **M)** Creatine quantification in iWAT from mice treated as described in L) after 6 hours of cold exposure. **N)** Effect of the creatine antagonist β-GPA on cold response in mice treated with SANA. **O)** Electromyogram during cold exposure.

In line with the proteomic signature, thermal images showed that SANA-treated mice had increased surface temperature, not only in their backs, but also in the interscapular space, corresponding to brown adipose tissue (BAT, Fig. 5A). Tissue microscopic analysis in iWAT showed an appearance consistent with a beigeing phenotype triggered by SANA (Fig. 5B). Indeed, SANA stimulated 18FDG uptake *in vivo* in iWAT **(Supplementary Fig. 5)**. Gene expression profiles in iWAT, showed that SANA stimulated the expression of *Ckmt1* as well as *Ckm* (Fig. 5C). Other genes involved in creatine metabolism, transport and synthesis including *Ant1*, *Gatm, Oat, Prodh and CrT* were up-regulated by SANA (Fig. 5C). *Ucp1* was down-regulated in mice treated with SANA when measured by mRNA (Fig. 5C), but no significant changes were observed by western blot (Fig. 5D). The thermogenesis-related genes *Cidea, Prdm16 and Pgc1ɑ* were also up-regulated by SANA (Fig. 5C). Importantly, treatment of differentiated human white adipocytes with SANA stimulated the expression of regulators of creatine metabolism, including CKMT1 and CKMT2 (Fig. 5E, **left**), as well as mitochondrial respiration (Fig. 5E, **right**). SANA also stimulated the expression of genes involved in creatine metabolism and thermogenesis in brown adipose tissue (BAT). Different from iWAT, the creatine kinases up-regulated by SANA in BAT were *Ckm* and *Ckmt2*. Other thermogenesis-related genes, like *Cidea*, *Pgc1ɑ*, *Gatm* and *Prodh* were up-regulated in BAT, whereas *Ucp1* was not altered (Fig. 5F). These changes in gene expression profile in BAT were reflected by increased creatine (Fig. 5G) and creatine kinase activity in isolated mitochondria (Fig. 5H). Measurements of respiration in isolated mitochondria showed that SANA increased state II respiration (Fig. 5I). However, BAT mitochondrial respiration inhibited by GDP (Fig. 5J) and stimulated after titration with oleate (Fig. 5K), attributed to UCP1 activity^21^, was not altered by SANA, supporting that increased state II respiration was not dependent on UCP1 regulation. Direct addition of SANA to BAT isolated mitochondria also had no effect on GDP-dependent respiration **(Supplementary Fig.5).** To further support the thermogenic potential of SANA, we performed cold challenge experiments. Mice treated with SANA (20 mg/kg SC, for 5 days) exposed to cold showed improved thermogenic response compared to controls (Fig. 5L), which was reflected in significantly increased creatine levels in iWAT (Fig. 5M) and up-regulation of creatine kinases and thermogenic markers (**Supplementary Fig.5**). To confirm that the improved response to cold was dependent on creatine, mice were treated with the creatine depleting drug β-guanidinopropionic acid (β-GPA)^22^, which can only be used acutely as chronic exposure can induce weight loss^20,22^. Mice were treated with SANA (20 mg/kg/day, SC) or SANA+β-GPA daily (0.4 mg/kg/day, IP) for 5 days. No changes in body weight or food consumption were detected among treatments (**Supplementary Fig. 5**). Treatment with β-GPA abolished the effect of SANA on thermogenic response, confirming that creatine is necessary for the effect of SANA on thermogenesis (Fig. 5N). Muscle activity was also assessed during cold exposure. Electromyographic analysis showed that shivering thermogenesis was not affected by SANA (Fig. 5O). Since creatine is a major muscle metabolite, we tested the potential for SANA to affect muscle function. Aerobic muscle capacity was performed and revealed that SANA did not impact muscle physiology (**Supplementary Fig. 5**). Creatine kinase expression and mitochondrial function in skeletal muscle and cells was not affected by SANA, although there was a significant increase in muscle creatine levels **(Supplementary Fig. 5).** Furthermore, treatment with SANA did not generate any observable deleterious effects on cardiac function and had no effect on heart mitochondrial function (**Supplementary Fig. 5**).

### SANA stimulates energy expenditure and protects against obesity under thermoneutral conditions.

**Figure 6.**
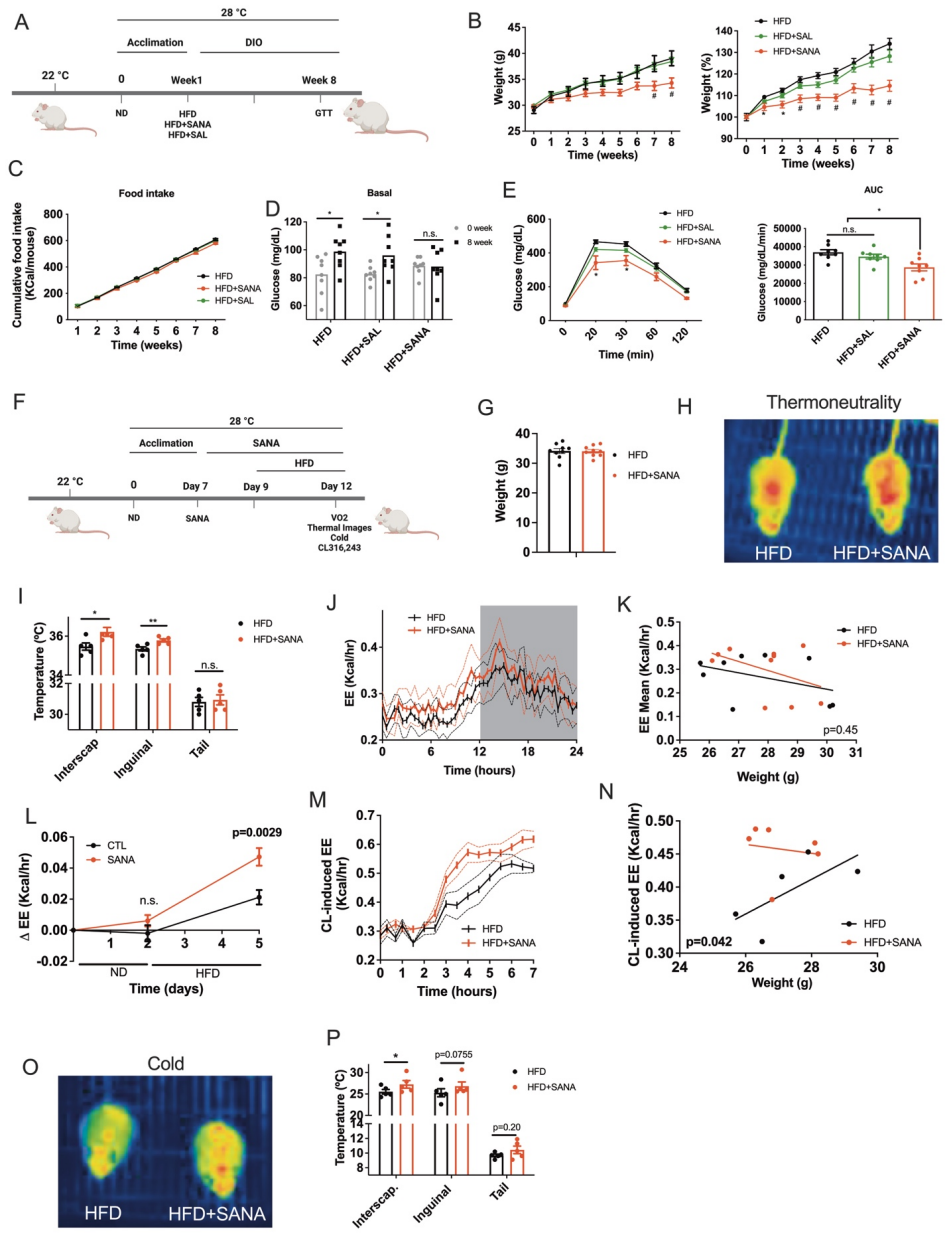
SANA stimulates energy expenditure and protects against obesity under thermoneutral conditions. **A)** Schematic representation of long-term treatment under thermoneutral conditions. Mice were fed with HFD or HFD+SANA (or SAL) at 20 mg/kg/day, SC. **B)** Weight gain and percent of initial weight. **C)** Cumulative food intake **D)** Fasting glycemia at weeks 0 and 8. **E)** GTT at week 8 **F)** Schematic representation of acute HFD and SANA treatment under thermoneutral conditions. **G)** Body weight and **H)** Representative thermal image of HFD and HFD+SANA treated mice at the end of the acute treatment. **I)** Surface temperature quantitation from thermal images. **J)** EE measurements over a 24-hour period at the end of the acute treatment. **K)** Regression plot of EE shown in J. **L)** Change in individual EE during HFD treatment at 28°C. **M)** EE measurement of CL316,243-treated mice injected at the end of the acute treatment. **N)** Regression plot of EE shown in M). **O-P)** Thermoneutral-to-cold challenge switch of mice at the end of the acute treatment. **O)** Representative thermal image after 1 hour incubation at 4°C. **P)** Surface temperature quantitation from thermal images.

One caveat of envisioning thermogenesis as a suitable approach to treat obesity in humans is that they spend most of their time under thermoneutral conditions. Interestingly, disruption of creatine metabolism in adipose tissue decreases energy expenditure and promotes obesity in thermoneutrality^23,24^. Administration of SANA under thermoneutral conditions (Fig. 6A) also protected against DIO (Fig. 6B), with no effect on accumulated food intake (Fig. 6C). SANA was also effective in preventing diet-induced hyperglycemia (Fig. 6D) and glucose intolerance (Fig. 6E), further supporting that SANA may be a suitable treatment for obesity under thermoneutral conditions. To clearly determine that increased energy expenditure due to thermogenesis is a driving force for the protection against obesity but not a side effect of differences in body weight, we followed a protocol of acute HFD feeding (Fig. 6F) as previously described^24^. During this short treatment there was no difference in weight gain among groups (Fig. 6G). However, thermal imaging showed that SANA promoted heat dissipation (Fig. 6H–I). Consistently, mice treated with SANA during acute HFD feeding showed a slight but consistent increase in energy expenditure (EE) (Fig. 6J–K) that became statistically significant when the difference between basal (before HFD) and HFD-stimulated EE was calculated (Fig. 6L). These results are consistent with previous reports showing that the HFD is necessary for activation of creatine metabolism and energy expenditure in adipose tissue^24^. Indeed, treatment of mice with SANA fed with normal chow showed no changes in EE **(Supplementary Fig. 6).** To further confirm our results, we administered the β3-adrenergic agonist CL316,243 after 3 days of acute HFD feeding. Single treatment with CL316,243 stimulated a transient increase in EE, which was significantly increased in SANA-treated mice, irrespective of body weight, as shown by ANCOVA analysis (Fig. 6M–N). Additionally, after acute HFD feeding at 28°C, the mice were exposed to cold and surface temperature was assessed. Mice treated with SANA showed increased heat dissipation measured by thermal imaging (Fig. 6O–P).

### Unbiased search for SANA-binding proteins followed by *in vivo* confirmation identified CKMT1/2 as putative targets of SANA.

**Figure 7.**
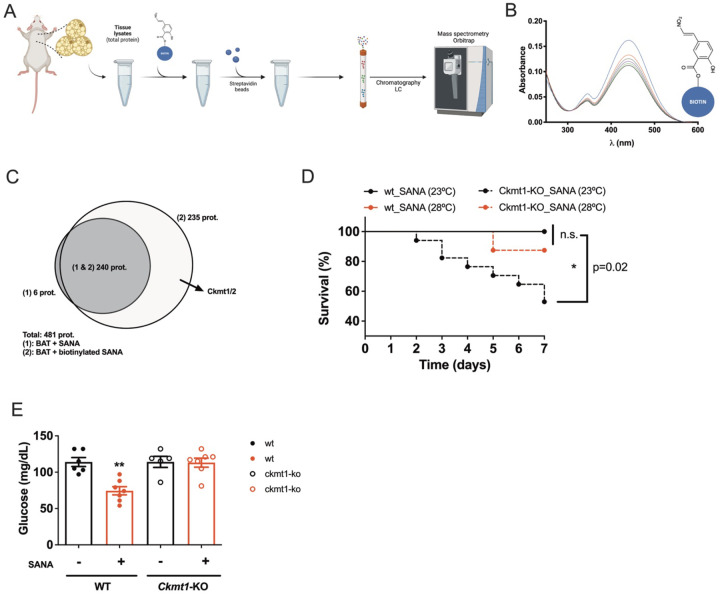
Identification of SANA-binding proteins followed by *in vivo* confirmation identified Ckmt1/2 as putative targets of SANA. **A)** Workflow scheme describing the screening strategy for the identification of SANA-binding proteins. **B)** Electrophilic properties of biotinylated SANA **(**bSANA). bSANA (10 μM) was incubated with β-mercaptoethanol (BME, 100 μM). Spectra of the reaction were obtained in the 200–600 nm range every 60s. **C)** Venn diagram showing proteins that were identified bound to SANA (control) or bSANA. **D)** Survival curve of WT and *Ckmt1* KO mice treated with SANA (20 mg/kg/day, SC) for 1 week at RT or thermoneutrality. **E)** Fasting glucose in WT and *Ckmt1* KO mice at thermoneutrality after HFD feeding and treatment with SANA (20 mg/kg/day, SC) or vehicle for 1 week.

Finally, we identified putative targets of SANA by unbiased pull-down of SANA-binding proteins followed by MS protein identification (Fig. 7A). We designed and synthesized a biotin-bound SANA (bSANA), maintaining its reactive nitroalkene function (Fig. 7B) and seek for SANA binding proteins in BAT. MS analysis showed that bSANA bound to ~300 proteins. Interestingly, CKMT was among the top proteins bound to bSANA (Fig. 7C **and Supplementary Table VII**). The high level of similarity between CKMT1 and 2 did not allow for complete discrimination by MS **(Supplementary Table VII).** Interestingly, *Ckmt1 and Ckmt2* are up regulated by SANA in iWAT and BAT, respectively (Fig. 5) and during cold challenge in BAT **(Supplementary Fig. 7)**. Importantly, both C*kmt1 and* C*kmt2* are up-regulated (together with *Ckb*) upon forskolin treatment in differentiated human brown adipocytes *in vitro*, and both kinases (*Ckmt1* and *Ckmt2*) are up-regulated during cold exposure *in vivo* **(Supplementary Fig. 7).** Finally, we tested the effect of SANA on *Ckmt1* KO mice during short-term HFD. To our surprise, we found that when mice were kept at room temperature (RT), *Ckmt1* KO mice showed ~50% of mortality rate after SANA treatment. No mortality occurred in WT mice (Fig. 7D). The effect on mortality was rescued when *Ckmt1* KO mice were acclimated at thermoneutrality during the treatment with SANA, showing that SANA is functionally interacting with CKMT1 and affecting thermoregulation *in vivo*. Additionally, the glucose lowering effect of SANA at thermoneutrality was lost in *Ckmt1* KO mice (Fig. 7E), further supporting the role of CKMT1 in SANA-driven effect.

## Discussion

Obesity and its metabolic complications are a challenging problem for pharmaceutical interventions. The multifactorial causes of obesity, and the need for long-term interventions with associated side effects highlight the necessity for constant pharmacological innovation. The irruption of the GLP-1 mimetics provides hope for effective and safe treatments for obesity^25^. However, the massive use of GLP-1 agonists is under pharmacological surveillance, and evidence about long term safety, efficacy and compliance are still being collected. In that sense, developing alternative and even complementary approaches is of need. The use of novel hybrid drugs based on well-designed modifications of safe and inexpensive compounds may provide a suitable way to provide massive, safe, and affordable therapies for this pandemic disease.

The current study addressed the effects of a derivative of the ancient and long-time used drug salicylate, 5-(2-nitroethenyl) salicylic acid, on the prevention and treatment of DIO and its related metabolic abnormalities. The data demonstrate that: 1) SANA is effective for weight loss and weight gain prevention, 2) the primary metabolic abnormalities associated with obesity are improved by SANA, 3) the beneficial effects of SANA are substantially greater than those obtained with salicylate 4) the effects of SANA are mediated by stimulation of adipose tissue mitochondrial respiration, 5) SANA protects against DIO at thermoneutral conditions, situation closely related to normal human environmental conditions, and 6) a primary target of SANA is creatine metabolism in adipose tissue and non-shivering thermogenesis, through a direct binding and regulation of CKMT1/2.

Several reports have shown the salutary effects of salicylate in DIO^3–5,26^ as well as novel important cell signaling properties^6,19^, promoting clinical trials aimed at using salicylate to treat chronic metabolic diseases. However, when studied in humans, the positive metabolic effects were somewhat disappointing, leading to a loss of impetus for investigating their potential clinical use. A further issue was the high doses required, resulting in the development of side effects^7–9^. Remarkably, SANA is effective at doses that are up to 40x lower than salicylate. Together, these data suggest that clinical trials with SANA have the potential for more beneficial metabolic outcomes at substantially lower doses and with reduced side effects. Activation of non-shivering thermogenesis, a homeostatic mechanism present in all mammals, is an attractive approach for treating obesity^27^, with several reports showing that adipose tissue-dependent thermogenesis is stimulated in humans after cold exposures^28,29^. Salicylate promotes thermogenic activity in murine BAT by UCP1-dependent and UCP1 independent mechanisms^3–5,30^. Salicylate also stimulates shivering thermogenesis in murine skeletal muscle^5^. Our analysis of adipose tissue indicated that the salutary effects of SANA are also associated with stimulation of non-shivering thermogenesis. Importantly, the effects are mediated through an increase in creatine-related energy expenditure, reminiscent of creatine-dependent thermogenesis^31,32^. In fact, SANA stimulates thermogenesis independently of detectable UCP1 activation (See Fig. 5) and does not directly uncouple of mitochondria as was proposed for salicylate^4^. SANA activated several key regulatory genes that have been identified as requisite to boost creatine metabolism in adipose tissue, including different creatine kinases and key enzymes for creatine synthesis. Disruption of creatine metabolism *in vivo* completely abolished the pro-thermogenic effect of the drug. SANA binds to the mitochondrial creatine kinases CKMT1/2. Further, our results show that SANA-induced thermogenesis is dependent on this interaction, since CKMT1 KO mice had compromised thermoregulatory capacity when treated with the compound (Fig. 7). Furthermore, when maintained at thermoneutrality, CKMT1 KO mice showed no response to SANA in glucose management (Fig. 7). Some of us recently proposed that CKMT1 is dispensable for mitochondrial function in white adipose cells, and CKMT1 KO mice have no alterations in metabolic parameters in normal or HFD feeding^33^. The fact that *Ckmt1* KO mice have diminished thermoregulatory capacity when treated with SANA, suggests that CKMT1 plays a role in thermoregulation and that *Ckmt1* KO mice might develop compensatory mechanisms to cope with this deficiency. It also suggests that treatment with SANA somehow commits mice to creatine-dependent metabolism for thermoregulation, in detriment of other thermogenic pathways, compromising the flexibility of the thermogenic response. In fact, depletion of creatine *in vivo* not only abolished the effect of SANA on thermoregulation, but in some cases seemed to worsen it (Fig. 5N). Experimental evidence suggests the existence of a dynamic crosstalk between UCP1 and creatine-dependent thermogenic pathways where they seem to go in opposite directions^20^. It is plausible then, that hyperactivation of creatine metabolism in adipose tissue by SANA will impede the activation of UCP1 in case of need. This is an important issue to address and will deserve future investigation. Originally, CKMT1 was proposed to drive creatine phosphorylation in beige adipose tissue^20^. Recent findings from the same researchers proposed that CKB controls creatine-dependent thermogenesis in BAT^34^. However, the role of CKB in mitochondrial creatine metabolism^35^, and how creatine-dependent thermogenic cycle works has been questioned^36,37^. In human adipose cells CKB is exclusively located in the cytosol^35^, suggesting that the regulation of this pathway is complex and needs further investigation. On the other hand, several reports suggest that CKMT2 is an important player in adipose tissue thermogenesis, i.e.: downregulation of *Ckmt2* in adipose cells decreases phosphocreatine levels^35^; silencing of β3-adrenergic receptors in beige/brown adipose cells decreases both *Ckmt1* and *Ckmt2* expression^38^. Also, bile acids increase mitochondrial function in white adipocytes, stimulating a thermogenic program that is partially dependent on CKMT2 expression^39^. There is also evidence suggesting that downregulation of *Ckmt2* in skeletal muscle in hibernating bears is linked to metabolic reprogramming involving an increase in glycolysis^40^.

We found that in iWAT, SANA stimulated both *Ckmt1* and *Ckm* during DIO. However, in BAT, *Ckmt2* was the main kinase up regulated by SANA. While there is no evidence supporting that *Ckm* participates in creatine-dependent thermogenesis, the differential response among treatments and tissues suggests that there may be some level of redundancy among contributory kinases, as well as differential responses between acute and chronic thermogenic activity. In fact, RNAseq analysis of human adipocyte cells^41^ show that activation by forskolin promotes an increase in expression of more than one creatine kinase, which is also seen *in vivo* by cold exposure (**Supplementary Fig. 7**). Interestingly, an alternative model for creatine-dependent thermogenesis was recently proposed, mirroring its role in skeletal muscle, as a fast energy provider for Ca^2+^ reuptake into the endoplasmic reticulum^36^. Under this proposal, two kinases, one mitochondrial and one cytosolic are necessary, and is compatible with our results with SANA, where *Ckm*, is systematically up-regulated. Interestingly, cold exposure promotes the increase of mitochondrial and cytosolic creatine kinases both in iWAT and BAT **(Supplementary Fig. 7).** A thermogenic model involving two kinases would also explain why both CKMT1 and CKB were sequentially proposed as regulators of creatine-dependent thermogenesis^20,34^. However, shedding light into this issue goes beyond the scope of this work and will certainly be part of future research. Furthermore, if SANA completely recapitulates the creatine-dependent thermogenesis pathway that is activated during cold exposure needs further investigation. In fact, when we studied cold response in BAT and iWAT in untreated mice, we also found that Ckb is up-regulated (**Supplementary Fig. 7**), despite this creatine kinase is not affected by SANA. It is plausible then, that the pharmacological pathway activated by the drug phenocopies the physiological thermogenic, cold-elicited creatine-dependent thermogenesis, but through activation of different kinases.

In summary, the current study presents compelling evidence that a nitroalkene-salicylate (SANA) is a potent drug for the treatment of diet-induced obesity, with a primary mechanism of action being the activation of creatine-dependent energy expenditure and thermogenesis in adipose tissue. Importantly, this activation also occurs at thermoneutrality, a condition that reflects everyday human life. This report provides the first evidence that boosting creatine metabolism in adipose tissue is an effective pathway to prevent and treat DIO. These observations reinvigorate the possibility that chemical derivatives of salicylates may be suitable drugs for the treatment of obesity and associated metabolic abnormalities. Confident on this possibility, we are now conducting a FIH clinical trial with SANA (medical name for the trial is MVD1: “A Phase 1, Randomized, Double-blind, Placebo-controlled, First in Human Study of the Safety, Tolerability, Pharmacokinetics and Pharmacodynamics of single and multiple doses of MVD1 in healthy adult volunteers”).

## Conclusions

Non-shivering thermogenesis is a homeostatic response present in mice and humans that can be subject of pharmacological stimulation. Boosting of creatine metabolism and thermogenesis in adipose tissue reveals a novel therapeutic opportunity for the treatment of metabolic diseases, including obesity, NAFLD, metabolic syndrome and type II diabetes. SANA is a potent activator of this pathway, revealing significant potential for clinical application. The feasibility of SANA as a drug for human consumption will depend on its safety and efficacy in patients. However, decades of accumulated data on the pharmacological properties and salutary actions of salicylate combined with preclinical data on SANA actions in mice lay the fertile foundation for further clinical development.

## Methods

### Animals

C57BL/6J mice and zebrafish used in this study were maintained at the Institut Pasteur de Montevideo Animal facility (UBAL). The experimental protocol was approved by the Institutional Animal Care and Use Committee of the Institut Pasteur de Montevideo (CEUA, Protocol numbers 003–19 and 006–19). Studies were performed according to the methods approved in the protocol. All the experiments were performed on adult male mice (10–12 weeks of age) and were conducted at either 22°C or 28°C (thermoneutrality) with free access to food and water. Unless specified, mice were housed at 22°C in groups of five. For thermoneutrality experiments, mice were housed individually at 28°C for one week before starting the experiment.

For the CLAMS studies, C57BL/6J mice were purchased from the Jackson Laboratory, and the experiments were approved by the Institutional Animal Care and Use Committees of West Virginia University

Ckmt1 null (KO) and wild-type (WT) mice on a C57BL/6N background were maintained at the University of Guelph as previously described^33^. All experiments were approved by the Animal Care Committee at the University of Guelph and met the guidelines of the Canadian Council on Animal Care.

### Drugs delivery

SANA (or salicylate) was administered orally (PO) by mixing with the food. For oral gavage (SANA, salicylate, and metformin) or subcutaneous (SC) (SANA, M1: 5-(2-nitroethyl)salicylic acid) administration, drugs were prepared in a 100 mM phosphate buffer, pH 6.5/ PEG 400 (50/50; v/v). In all cases the vehicle was used as control.

### High-fat diet feeding

Male C57BL/6J mice housed at 22°C or 28°C were fed a high-fat diet (42% fat and 0.25% cholesterol, AIN93G, LabDiet, USA), starting at 12 weeks of age and for a duration of 8–20 weeks.

### Acute high-fat diet feeding

Individually-housed C57BL/6J male mice were acclimated for one week at 28°C and fed with normal chow. At that point, SANA (20 mg/kg/day, SC) administration was started and repeated for 5 days. On the second day of administration, the normal chow diet was changed to a high-fat diet for the subsequent three days. In all cases the vehicle was used as control.

### Cumulative food intake

Intake was measured individually once per week for a 24 h period and expressed as total calories per mouse, independent of body weight.

### Cold challenge experiments

C57BL/6J male mice previously acclimated at 22°C were treated or not (control) with SANA (or SAL, 20 mg/kg/day, SC), in combination or not with beta-guanidinopropionic acid (β-GPA, 0.4 g/kg in PBS pH 7.5, IP) for 5 days and fed with normal chow. At that point, mice were housed individually at 4°C from one to six hours, with free access to food and water. Body temperature was measured before and during the challenge (once every hour) with a mouse rectal probe (ThermoWorks).

### Thermal imaging

Thermal images were collected from non-anesthetized, awake mice with a FLIR E6 thermal imaging camera. Regions of interest from thermal images were selected and quantified using the FLIR Tools software.

### Blood glucose measurements

Mice were kept in fasting for 16 h before fasting glucose measurement and glucose tolerance tests (GTT). For GTT mice were injected (IP) with 1.5 g/kg body weight of glucose solution. Plasma glucose concentrations were measured from blood obtained from the tail using a hand-held glucometer (Accu-Chek, Roche).

### Insulin, leptin, FFA and liver transaminases measurements

Quantitation of insulin (Mouse INSULIN ELISA Kit, Thermo Sc.); non-esterified fatty acids (NEFA, NEFA-HR Assay, Waco); leptin (Mouse leptin ELISA Kit, Abcam) and hepatic transaminases (Liver & Kidney Profile, MNCHIP) were performed in plasma/serum following the supplier’s instructions.

### Comprehensive Lab Animal Monitoring System (CLAMS)

Mice were maintained in the CLAMS (Columbus Instruments, Columbus, OH, USA) for 13 days at 28 °C. They were acclimated for 7 days and fed with normal chow. At that point, SANA administration (20 mg/kg/day, SC) was started and repeated for 6 days. On the second day of administration, the normal chow diet was changed to a high-fat diet for the subsequent four days. On the last day mice were injected with CL 316, 243 (1 mg/kg, IP in saline solution). In all cases the vehicle was used as control. Oxygen consumption (VO2), carbon dioxide production (VCO2), and activity of individual mice were monitored over the total period. VO2 and VCO2 values were used to calculate respiratory exchange ratio (RER), and VO2 and RER values were used to determine energy expenditure (kcal/h).

### Body composition

Lean mass and fat mass of individual mice were measured by quantitative NMR using an EchoMRI analyzer (Houston, TX, USA).

### Chemistry

Chemicals were purchased at the highest purity available. ^1^H NMR and ^13^C NMR spectra were recorded on a Bruker DPX-400 instrument, with DMSO-d6 as solvent and tetramethylsilane as the internal reference. Electron impact (EI) and electrospray (ES+) mass spectra were obtained at 70 eV on a Shimadzu GC-MS QP 1100 EX or on a Hewlett Packard 1100 MSD spectrometer, respectively. HRMS was obtained in a Q Exactive Plus mass spectrometer (Thermo Scientific, USA) by direct injection using 30% acetonitrile as solvent and an Ion Max API source with a HESI-II probe. The mass spectrometer was operated in a negative mode, ion spray voltage was set at 3.5 kV and capillary temperature at 250°C. TLC was carried out on Alugram^®^ Sil G/UV254 on polyester plates. All solvents were of anhydrous quality purchased from Aldrich Chemical Co. and used as received.

5-(2-nitroethenyl)salicylic acid (SANA) synthesis: To a solution of 5-formylsalicylic acid (19.2 g, 116 mmol) in ethanol (176 mL), nitromethane (24 mL, 443 mmol) and ammonium acetate (22.3 g, 144 mmol) were added. The reaction mixture was heated at 60°C for 2 h, an orange precipitate was observed. The reaction mixture was allowed to cool to room temperature and was acidified with concentrated HCl (~30 mL). The orange solid gives a yellow solid and it was filtered under vacuum. The solid is thoroughly washed with water to remove the ammonium chloride formed and recrystallized from water:ethanol (1:1) to give yellow crystals. mp. 230–232°C. Yield: 93%.

^1^ H NMR (400 MHz, DMSO-d6): δ= 8.26 (d, *J*=2.2 Hz, 1H), 8.21 (d, *J* 14.8 Hz, 1H), 8.18 (d, *J* 14.8 Hz, 1H), 8.04 (dd, *J* 8.7 2.2 Hz, 1H), 7.06 (d, *J* 8.7 Hz, 1H).

^13^ C NMR (101 MHz, DMSO-d6): δ= 171.6, 164.5, 139.2, 136.9, 136.2, 134.1, 122.0, 118.8, 114.6.MS (EI, 70eV): *m/z* (%) 209(M^+^, 65), 191(100), 162(23), 144(45), 122(20).

HRMS: m/z [M-H]^−^ calculated for C_9_H_7_NO_5_: 208.0251, found: 208.0247 ± 0.0002.

### iWAT mitochondria enrichment

For each biological replicate, subcutaneous inguinal white adipose tissues from 2–3 animals (according to body weight) were dissected, pooled, and washed in ice-cold homogenization buffer (HB) (250 mM sucrose, 10 mM HEPES, 0.1 mM EGTA, pH 7.2). Homogenization buffer was supplemented with 2% BSA during the isolation of fat mitochondria and the procedure was conducted at 4°C. Tissues were minced on ice and homogenized in HB (5 ml/g) using a motorized Potter-Elvehjem teflon pestle. Homogenates were filtered through clean gauze to remove fat particles and centrifuged at 1000 g for 5 min to pellet nuclei and cell debris. The supernatant was transferred to a clean tube and centrifuged at 10000 g for 15 min to pellet crude mitochondria. The mitochondrial pellet was washed three times with 2 mL of HB (without BSA) by centrifugation at 10000 g for 10 min and stored at −80 °C until used.

## Proteomics analysis

### Sample preparation for LC-MS/MS analysis

For proteomics analyses, three biological replicates were used per condition. Protein quantification was performed by densitometry analysis of whole iWAT or iWAT enriched mitochondrial samples separated on 12,5% SDS-PAGE gels and using a low molecular weight protein calibration kit (Amersham, GE Healthcare). For mass spectrometry analysis, equal amounts of each protein sample were separated only up to 1 cm into the resolving gel, fixed and stained with Coomassie blue R-250. In-gel protein digestion and peptide extraction were performed as previously described^42^ with minimal variations. Briefly, the 1 cm bands were excised, and cysteine residues were reduced and alkylated by sequential incubation with 10 mM dithiothreitol (DTT) and 55 mM iodoacetamide (IAA). Tryptic digestion was performed in-gel by overnight incubation at 37°C with sequencing grade trypsin (Promega) in a protease:protein ratio of 1:50 (w/w). Tryptic peptides were extracted from the gel by adding 60% ACN / 0.1% trifluoroacetic acid (TFA) in two steps of 1h incubation at 30°C. Samples were dried under vacuum and peptides were desalted using ZipTips C18 (Merck Millipore). Eluted peptides were vacuum dried and dissolved with 0.1% formic acid (FA).

### LC-MS/MS analysis

LC-MS/MS analysis was performed with an UltiMate 3000 nanoHPLC system (Thermo Fisher Scientific) coupled to a Q Exactive Plus mass spectrometer (Thermo Fisher Scientific, USA). Samples were loaded onto a precolumn (Acclaim PepMap^™^ 100, C18, 75 μm X 2 cm, 3 μm particle size) and separated with an Easy-Spray analytical column (PepMap^™^ RSLC, C18, 75 μm X 50 cm, 2 μm particle size) at 40°C using a two-solvent system: (A) 0.1% FA in water, (B) 0.1% FA in acetonitrile (ACN). Separation was achieved through an elution gradient as follows: 1% to 35% B over 150 min and 35% to 99% B over 20 min, at a flow rate of 200 nL/min. The mass spectrometer was operated in a positive mode using a top-12 data-dependent method. Ion spray voltage was set at 2.5 kV and capillary temperature at 250°C. The survey scans were acquired in a range of 200–2000 m/z with a resolution of 70000 at 200 m/z, an AGC target value of 1E6 and a maximum ion injection time of 100 ms. Precursor fragmentation occurred in an HCD cell with a resolution of 17500 at 200 m/z, an AGC target value of 1E4 and a maximum ion injection time of 50 ms. Normalized collision energy was used in steps of NCE 25, 30 and 35. Dynamic exclusion time was set to 30 s. Each sample was injected twice.

### Mass spectrometry data analysis

PatternLab for Proteomics 4.0 software (PatternLab)^43^ was used to perform peptide spectrum matching and label free quantitative analysis. For data search, a target reverse database was generated using PatternLab, including *Mus musculus* proteome (downloaded from UniProt, 26/08/2020) and the most common contaminants in proteomics experiments. For peptide identification m/z precursor tolerance was initially set at 40 ppm and methionine oxidation and cysteine carbamidomethylation were defined as variable and fixed modifications respectively. A maximum of 2 missed cleavages and 2 variable modifications per peptide were allowed. Search results were filtered by the PatternLab Search Engine Processor (SEPro) algorithm with a maximum FDR value ≤ 1% at protein level and 10 ppm tolerance for precursor ions. PatternLab’s Venn diagram statistical module was used to determine proteins uniquely detected in each biological condition using a probability value less than 0.05^43^. PatternLab’s TFold module was used to relatively quantify proteins present in both biological conditions by a spectrum count-based label-free quantification method. Proteins present in at least 4 biological replicates from the total of 6 were considered for TFold analysis. This module uses the Benjamini-Hochberg’s theoretical estimator to deal with multiple T-tests and it maximizes the number of identifications satisfying a fold change cutoff that varies with the p-values (BH q<0.05); while restricting false differential proteins mainly due to low abundance^44^. The list of statistically overrepresented proteins in the condition HFD+SANA, obtained from Venn diagram and TFold Patternlab’s modules, was subjected to pathway enrichment analysis. The *Mus musculus* total proteome or the mitochondrial proteome obtained in our experiment were used as background proteomes for the overrepresentation analysis of the total or mitochondrial protein sets respectively, using the functional enrichment analysis web tool WebGestalt^45^. The mass spectrometry proteomics data have been deposited to the ProteomeXchange Consortium via the PRIDE^46^ partner repository with the dataset identifier PXD030485 (for reviewer access: Username: reviewer_pxd030485@ebi.ac.uk, Password: tNnBLEHe).

### Metabolite analysis by LC-MS/MS and NMR

Subcutaneous adipose tissue samples (100 mg) were homogenized and extracted with an ice-cold mixture of equal volumes of water, methanol, and chloroform (1.0 mL). The aqueous phases were separated and dried in a SpeedVac, and the resulting tissue extracts were reconstituted in 600 μL of phosphate buffer (pH 7.4) containing 0.02% NaN3 and 20% D2O and transferred to 5 mm NMR tubes (NE-HL5–7, New Era Enterprises Inc., Vineland, NJ, USA). All NMR experiments were performed on a Bruker AVANCE III 500 NMR spectrometer equipped with a room temperature z-gradient TXI probe and operating at 1H and 13C frequencies of 500.13 and 125.76 MHz, respectively. Water-suppressed 1H NMR spectra were recorded using a 1D-NOESY pulse sequence with presaturation at 25 °C. A spectral width of 10 KHz, a data size of 32 K, and a total of 128 scans with a relaxation delay of 4 s between scans were employed to record each experiment. All free induction decays were zero-filled to 64 K points and apodized with a 0.3 Hz exponential window function prior to Fourier transformation. Spectra were referenced to the a-glucose anomeric proton resonance at 5.22 ppm present in all samples. Metabolites were identified by comparison to spectra from public databases, assisted with data obtained from standard gradient-enhanced HSQC spectra when necessary. Metabolite concentrations in the NMR sample were estimated using the PULCON method as implemented in the spectrometer (54) and normalized to wet tissue mass to yield concentrations in nmol/mg of tissue.

For LC-MS/MS analysis, dried tissue extracts were reconstituted in 200 μL of 5 mmol/L ammonium acetate in 95% water, 5% acetonitrile, and 0.5% acetic acid was passed through a 0.45-μm polyvinylidene fluoride filter. The extracts were analyzed by a Shimadzu LC Nexera X2 UHPLC coupled with a QTRAP 5500 LC-MS/MS (liquid chromatography/ mass spectrometry-mass spectrometry; AB Sciex). An ACQUITY UPLC BEH Amide analytic column (2.1×50 mm, 1.7 μm, Waters) was used for chromatographic separation. The extracted MRM peaks were integrated using MultiQuant 3.0.2 software (AB Sciex).

### Identification of SANA-binding proteins

To search for SANA targets, 100 mg of mouse brown adipose tissue were lysed in 500 μL of NETN buffer (20 mM Tris pH 8.0, 100 mM NaCl, 1 mM EDTA, 0.5% NP40) using a bullet blender. Homogenates were centrifuged at 10000 g for 10 min, 4°C, and the supernatant’s protein concentrations were measured by Bradford assay (PanReac AppliChem) using a BSA (Capricorn Scientific) standard. Four replicates of 200 μL of brown adipose tissue lysate containing 100 μg of total protein each, were incubated with 100 μM SANA or biotinylated-SANA at 25 °C for 30 min in agitation. A Strep-Tactin^®^ Sepharose^®^ resin (IBA Lifesciences) suspension in NETN buffer (50:50 v/v; 30 μL total volume) was added to each sample. The mixture was incubated in rotation for 1 h at 4°C and centrifuged at 1000 g for 1 min. The supernatant was discarded, and the resin was washed 4 times with 500 μL of NETN. The resin was resuspended in 60 μL of reducing SDS-PAGE sample buffer (62.5 mM Tris-Cl, 2% SDS, 10% v/v glycerol, 10% 2-mercaptoethanol, 0.01% bromophenol blue, pH 6.8) and heated at 95°C for 5 min. A fraction of 20 μL from each sample was loaded in a 1D SDS-PAGE and trypsin digested in-gel as described in “Sample preparation for LC-MS/MS analysis”.

The mass spectrometry analysis was performed as described in “LC-MS/MS analysis” except for the elution gradient: 1% to 35% B over 60 min and 35% to 99% B over 15 min; the spray voltage was set at 2.3 kV.

The mass spectrometry data analysis was performed with PatternLab 5.0 software^47^ using the same search parameters described for total and mitochondrial proteomes. The target reverse database was generated from a *Mus musculus* proteome (downloaded from UniProt, 08/04/2022) and the most common contaminants in proteomics experiments. PatternLab’s Venn diagram statistical module was used to determine proteins uniquely detected in each biological condition using a probability value less than 0.05, a minimum 2 replicates per condition to be considered, and the Lenient stringency mode^47^. PatternLab’s TFold module was used to relatively quantify proteins present in both biological conditions by a spectrum count-based label-free quantification method. Proteins present in at least 6 biological replicates from the total of 8 were considered for TFold analysis^44^. Proteins with a BH q<0.05 and a fold change greater than 1.5 were considered as significantly overrepresented in a biological condition. The list of statistically overrepresented proteins in the condition BAT + biotinylated SANA, obtained from Venn diagram and TFold Patternlab’s modules is listed in supplementary table VII. The mass spectrometry proteomics data have been deposited to the ProteomeXchange Consortium via the PRIDE^46^ partner repository with the dataset identifier PXD040949 (for reviewer access: Username: reviewer_pxd040949@ebi.ac.uk, Password: DKT6NzQ9).

### Electromyography (EMG)

Muscle electrical activity (EMG) was recorded in 4 C57Bl/6J adult male mice. A radiofrequency transmitter (EA-F20, Data Sciences International DSI, St. Paul, MN) with 2 wire leads was implanted subcutaneously under anesthesia with isoflurane (2.5% at 0.5 lpm O2) (protocol number: 70153-000673-18). The electrodes were sutured to the back muscles near the neck and the skin was closed with metal clips. Mice were allowed to recover for at least 72 h. Then, EMGs of the conscious restrained mice were recorded at a sampling frequency of 1000 Hz for 30 min in the morning (10–11 am) at 23°C and 4°C before (control) and after 5 day-treatment with SANA (20 mg/kg/day). Off-line analysis was performed to count shivering bursts in 5 minute-periods (30 windows,10 s each). A burst was counted if its duration was at least 200 ms^48^.

### Cell culture, differentiation, and treatments

TERT-hWA cells^49^ were cultured in Advanced DMEM/F12 (Life Technologies) supplemented with 10% FBS, L-glutamine (2 mM) (Life Technologies), penicillin (62.5 μg/ml), streptomycin (100 μg/ml) and basic fibroblast growth factor (bFGF) (2.5 ng/mL) (Life Technologies). TERT-hWAT preadipocytes were maintained as a subconfluent monolayer culture. Two days post-confluence (day 0), cells were induced to differentiate in Advanced DMEM/F12 supplemented with 2% FBS, L-glutamine (2 mM), penicillin (62.5 μg/ml), streptomycin (100 μg/ml), insulin (5 μg/ml), dexamethasone (1 μM) (Sigma- Aldrich), IBMX (0.5 mM) (Thermo Scientific), rosiglitazone (1 μM) (Sigma-Aldrich), human cortisol (1 μM) (Sigma-Aldrich) and T3 (1 nM) (Sigma- Aldrich). On day 3, the medium was refreshed with the same medium used at day 0. On day 6 and 9 of differentiation, IBMX, dexamethasone, insulin, rosiglitazone, and cortisol were omitted from the medium. At day 12, the adipocytes were considered mature.

### Isolation of IWAT adipocytes

Mice were fed with HFD and treated with SANA 100 mg/kg/day for 8 weeks. After that, mice were killed by cervical dislocation. Inguinal fat depots were dissected and weighed. Collagenase digestion solution (collagenase type II, GIBCO 1 mg/ml in Hank’s Balanced Salt Solution containing 2% BSA) was added in 3:1 collagenase fat tissue ratio, and tissue was fined minced and incubated for 1 hour at 37°C in a shaking incubator. Then adipocyte suspension was centrifuged at 30g, 3 minutes at RT. Floating mature adipocytes were washed twice with STEbuffer containing 2% BSA (250 mM sucrose, 5 mM Tris, 2 mM EGTA, pH = 7.4 at 4°C). Finally, adipocytes were suspended in MIR05 respiration buffer (0.5 mM EGTA, 3 mM MgCl2•6H2O, 60 mM MOPS, 20 mM taurine, 10 mM KH2PO4, 20 mM HEPES, 110 mM sucrose, 1 g/L BSA FA-free, pH 7.1.) with 2% free fatty acid BSA.

### Respirometry of adipocytes and cells

Oxygen consumption of cultured differentiated adipocytes and iWAT adipocytes were measured with high-resolution respirometer (Oxygraph-2k, OROBOROS INSTRUMENTS; Innsbruck, Austria) and Seahorse. Respiration measurements of C2C12 and SH-SY5Y cells were recorded in serum free DMEM media with additions of oligomycin (1 μg/mL), titration with carbonyl cyanide 4-(trifluoromethoxy) phenylhydrazone (FCCP, 0.5 μM steps) and antimycin A (0.5 μM) and rotenone (0.5 μM) for non-mitochondrial respiration. Adipocyte suspension was pipetted into 2.2 mL mitochondrial respiration medium (MIR05). For oxygen consumption measurements of intact adipocytes, pyruvate (5 mM) was injected, and basal cellular respiration was recorded (Schottl et al., 2015). Proton leak respiration was assessed by addition of oligomycin (2.3 μg/mL). Titration of carbonyl cyanide 4-(trifluoromethoxy) phenylhydrazone (FCCP, 0.5 μM steps) was performed for measuring maximal cellular respiration rates. Non mitochondrial oxygen consumption was determined in the presence of antimycin A (0.5 μM) and rotenone (0.5 μM). Adipocyte respiration measurements were normalized by the μg/mL of DNA. Basal respiration was determined before the addition of inhibitors and the uncoupler. Oligomycin-resistant and oligomycin-sensitive respiration rates were the ATP-independent (leak) and ATP-dependent respiration rates, respectively. Maximum respiration rate was obtained after titration with FCCP, and the Spare respiratory capacity was the difference between the Maximum and Basal respiration^50^.

Mito-Stress Test assays were performed using the Seahorse XFe96 Bioanalyzer and Seahorse 96-well XF cell culture microplates (Agilent Technologies, Santa Clara, CA). TERT-hWAT cells were plated and differentiated in seahorse plates. After differentiation cells were treated with or without SANA (100 μM) for 24 hs and after treatment cell culture media was exchanged for Mito-Stress assay media containing glucose (10 mM), glutamine (2 mM) and pyruvate (1 mM). This process was completed through an initial 100 μL assay media wash followed by suspension of 175 μL to each well. Real-time oxygen consumption and extracellular acidification rates (OCR and ECAR, respectively) were measured under basal conditions followed by sequential injections of forskolin (10 μM), oligomycin (2 μM), FCCP (1 μM), rotenone/antimycin A (1 μM).

### Brown adipose tissue mitochondria isolation

Brown adipose tissue (BAT) was dissected from the subscapular region, placed in tube containing ice cold isolation buffer (225 mM Mannitol, 75 mM Sucrose, 1 mM EGTA, 10 mM Hepes and 0,1% BSA fatty acid free – pH 7.4), and minced with a scissor. It was then disrupted in an ice-cold glass/Teflon Dounce homogenizer with the isolation buffer, filtered to remove the excess of fatty and transferred to a centrifuge tube. It was centrifuged at 1753 XG for 3 min at 4°C, the supernatant was discarded, and the pellet resuspended with the isolation buffer and transferred to a new centrifuge tube. It was centrifuged at 15777 XG for 10 min at 4°C, the supernatant was discarded, and the pellet resuspended with the isolation buffer and 15% of Percoll solution. It was centrifuged in a Percoll (Invitrogen) gradient (15%/23%/40%) at 12000 XG for 5 min at 4°C, acceleration 8 and deceleration 6. The layer formed between 23% and 40 % Percoll was collected, resuspended in the isolation buffer, and centrifuged at 12000 rpm for 10 min at 4°C with acceleration 8 and deceleration 6. The supernatant was discarded, and the pellet resuspended in 200 μL of respiration buffer MIR05 without BSA (MIR 05: 0,5Mm EGTA, 3mM MgCL2, 60 mM MES, 10mM KH2PO4, 10Mm HEPES, 110 mM sucrose – pH7.4).

### UCP1 activity of brown adipose tissue isolated mitochondria

Mice were treated for 15 consecutive days with SC injections of SANA (20 mg/kg/day) and vehicle. Brown adipose tissue mitochondria were isolated as before. (Oroboros O2K was used to perform a high resolution respirometry by the addition of 1 mM pyruvate, 1 mM malate, 1 mM ADP, 1 mg/mL BSA fatty acid free, 3 mM GDP and titration of 30, 60 and 90 μm oleate. The result was normalized by the protein concentration in the chamber. UCP1 activity is measured as sensitive respiration to GDP, an UCP1 inhibitor.

### Analysis of the electrophilic properties of SANA and biotinylated SANA (bSANA)

SANA (10 μM) or bSANA (10 μM) were dissolved in phosphate buffer 20 mM, pH 7.4 and incubated with β-mercaptoethanol (BME) or glutathione (GSH) 100 μM. The reaction was monitored by UV–Vis spectrophotometry through continuous absorbance readings (200–600 nm) every min during the first 10 min of the reaction using a Varian Cary 50 bio UV–VIS spectrophotometer (Agilent Technologies, Palo Alto, CA, USA).

### Creatine kinase activity

Forward and reverse creatine kinase activity was measured as described previously^51^. The forward reaction (production of phosphocreatine) was determined by oxidation of NADH to NAD+ at 340 nm, coupled to pyruvate kinase and lactate dehydrogenase. The assay buffer contained 5 mM ATP, 50 mM creatine, 130 mM KCl, 6 mM MgCl2, 0.4 mM PEP, 15 U/mL LDH, 7 U/mL PK and 15 mM Tris pH 8.8. For the reverse reaction (production of ATP), the activity was determined by NADH formation, coupled to hexokinase and glucose-6-phosphate dehydrogenase. The assay buffer included 1.5 mM ADP, 2 mM AMP, 50 mM DiAPP, 5 mM glucose, 130 mM KCl, 1 mM MgCl2, 0.7 mM NADP+, 9 mM phosphocreatine, 0.5 U/mL glucose-6- phosphate dehydrogenase, 1.3 U/mL hexokinase, and 10 mM Tris pH 7.4. The production of NADH was measured fluorometrically at an excitation wavelength of 340 nm and an emission wavelength of 460 nm. The rate was taken from the linear portion of the curve.

### Statistical analysis

Two ways ANOVA followed by Tukey’s multiple comparisons test or Ordinary one-way ANOVA followed by Bonferroni post hoc or unpaired Student’s t-test or linear regression and ANCOVA for in vivo metabolic analyses were used to calculate P values. In all cases * indicates p<0.05, ** indicates p<0.01, *** indicates p<0.001 and **** p<0.0001. In all cases, a minimum of five mice were used in each experimental condition. All data is presented as mean ± SEM. Calculations were done using GraphPad Prism.
